# Role of Circulating miRNAs in Therapeutic Response in Epithelial Ovarian Cancer: A Systematic Revision

**DOI:** 10.3390/biomedicines9101316

**Published:** 2021-09-26

**Authors:** Gloria Ravegnini, Pierandrea De Iaco, Francesca Gorini, Giulia Dondi, Isabella Klooster, Eugenia De Crescenzo, Alessandro Bovicelli, Patrizia Hrelia, Anna Myriam Perrone, Sabrina Angelini

**Affiliations:** 1 Department of Pharmacy and Biotechnology, University of Bologna, 40126 Bologna, Italy; francesca.gorini3@unibo.it (F.G.); patrizia.hrelia@unibo.it (P.H.); s.angelini@unibo.it (S.A.); 2 Division of Oncologic Gynecology, IRCCS Azienda Ospedaliero-Universitaria di Bologna, 40138 Bologna, Italy; pierandrea.deiaco@unibo.it (P.D.I.); giulia.dondi@aosp.bo.it (G.D.); eugenia.decrescenzo2@unibo.it (E.D.C.); alessandro.bovicelli@unibo.it (A.B.); myriam.perrone@aosp.bo.it (A.M.P.); 3 Department of Medical and Surgical Sciences, DIMEC, University of Bologna, 40138 Bologna, Italy; 4 Centro di Studio e Ricerca delle Neoplasie Ginecologiche, University of Bologna, 40138 Bologna, Italy; 5 Department of Pathology, Brigham and Women’s Hospital, 75 Francis Street, Boston, MA 02115, USA; iklooster@bwh.harvard.edu

**Keywords:** ovarian cancer, epithelial ovarian cancer, liquid biopsy, circulating miRNAs, drug response, personalized medicine, chemotherapy

## Abstract

Epithelial ovarian cancer (EOC) is one of the most lethal cancers worldwide, mostly due to nonspecific symptoms and a lack of screening tests, which, taken together, contribute to delayed diagnosis and treatment. The current clinical biomarker is serum CA-125, which allows the identification of most advanced primary and relapsed disease and correlates with disease burden; however, as well highlighted in the literature, CA-125 often lacks sensitivity and specificity, and is not helpful in monitoring chemotherapeutic response or in predicting the risk of relapse. Given that, the identification of novel biomarkers able to foster more precise medical approaches and the personalization of patient management represents an unmet clinical requirement. In this context, circulating miRNAs may represent an interesting opportunity as they can be easily detected in all biological fluids. This is particularly relevant when looking for non-invasive approaches that can be repeated over time, with no pain and stress for the oncological patient. Given that, the present review aims to describe the circulating miRNAs currently identified as associated with therapeutic treatments in OC and presents a complete overview of the available evidence.

## 1. Introduction

### 1.1. Epithelial Ovarian Cancer

Ovarian cancer represents the gynecological malignancy responsible for the highest number of deaths each year in western countries [1]. Ovarian cancers are a heterogeneous group of tumors including separate entities and are divided into epithelial (about 90% of cases), germ cell (3%), and sex cord–stromal (2%) [2]. Epithelial ovarian cancers (EOCs) are the most common and are, in turn, divided into serous ovarian carcinoma (SOC), endometrioid carcinoma (EMOC), clear cell carcinoma (CCOC), mucinous carcinoma (MCOC), Brenner tumors, undifferentiated, and carcinosarcomas. These cancers are grouped into a dualistic model, type I, and type II, which reflects different clinical–molecular features. Usually, type I tumors have indolent behavior and they are often limited to the ovary at the time of diagnosis; they have a stable genome with no *TP53* mutations, even if somatic alterations can be frequently detected in different genes, such as *BRAF*, *KRAS*, and *CTNNB1*. Type I tumors include low-grade SOC, EMOC, MCOC, CCOC, and Brenner tumors. On the other hand, type II tumors are more aggressive, usually identified at an advanced stage, and genetically highly unstable; the majority of them harbor *TP53* mutations, and a good portion of the cases have mutations or dysfunction of *BRCA-1/2* genes. High-grade serous EOC is the predominant histotype in type II cancers [3,4]. EOC is a relatively rare condition with the highest incidence rates in western countries such as in Europe and North America (8 cases per 100,000 population) [5,6]. This cancer is the most lethal and silent gynecological tumor with diagnosis in an advanced stage in about 80% of cases and a 5-year relative survival of only 20–30%. Primary tumors originate from the epithelium of the ovary, fallopian tube, or peritoneum and then spread to the peritoneal surface and to the viscera of the pelvis and abdomen (carcinosis). The standard approach is surgical cytoreduction followed by standard first-line chemotherapy with platinum and taxane compounds. When surgery is not feasible due to the extent of the disease, neoadjuvant chemotherapy is an option to reduce the burden of the disease and give the patient the opportunity for cytoreduction [7]. Despite optimal surgery and proper chemotherapy, approximately 70% to 80% of patients will develop a recurrent disease and gradually patients susceptible to platinum experience shorter intervals without illness, with the development of platinum resistance and poor prognosis [8,9,10,11]. In the last decade, targeted therapies including vascular endothelial growth factor (*VEGF*) inhibitors and poly (adenosine diphosphate-ribose) polymerase (*PARP*) inhibitors have been introduced with positive outcomes in clinical trials, but their role in OC therapeutic algorithms is still under debate [12].

Currently, serum markers (CA-125 and HE4) represent the only weapon to assess response to therapy, tumor progression, and disease recurrence. HE4, although of great promise, is not yet widely used in clinical practice because it has a clinical value overlapping with CA-125 [13]. Thus, serum CA-125 is the only biomarker available, but it lacks sensitivity and specificity that do not allow routine use for early diagnosis even in combination with other tools such as ultrasound [14]. It should be noted that a part of ovarian carcinomas is also CA-125 silent. It remains useful in the gross evaluation of response to therapy and its increase in treated patients is often a sign of disease recurrence. However, CA-125 assay does not correlate with the prediction of platinum sensitivity or resistance. In the absence of specific markers, presently, response therapy is evaluated surgically by diagnostic laparoscopy on the accuracy of laparoscopy to assess peritoneal spread in ovarian cancer [15].

### 1.2. Liquid Biopsy 

Currently, the gold standard approach for the histological diagnosis and genetic–molecular characterization of EOC is tissue biopsy; however, standard biopsy is an invasive procedure that provides a static picture of the disease, strictly related to the portion of tissue analyzed. Given its invasiveness, it cannot be repeated easily over time, thus, not providing it does not provide a reliable, dynamic image of tumor evolution. An appealing alternative approach attempting to overcome these limits is liquid biopsy, which allows the detection of circulating molecules directly released by the tumor mass in body fluids. Not surprisingly, in recent years there has been a growing research interest in this field. Liquid biopsy allows access, through a non-invasive approach, to molecular information or identifies specific biomarkers in biological fluids (including, but not limited to, blood, ascitic fluid, urine, saliva), which could be very helpful in better characterizing a cancer patient [16,17]. 

In the last decade, research advances have boosted several steps forwards, promoting in 2016 the FDA approval of the first diagnostic test based on liquid biomarkers for non-small cell lung cancer (NSCLC) [18,19]. This test can detect specific *EGFR* mutations in the blood of NSCLC patients, who would not be able to provide a tumor biopsy for conventional EGFR testing due to advanced tumor stage, comorbidities, or tissue inadequacy. This fosters the choice of the most suitable treatment, maximizing the benefits for those patients. As demonstrated by this, much progress has been directed to diagnosis; however, another important potential application of liquid biopsy is therapeutic monitoring in order to achieve more personalized treatment [20,21,22].

### 1.3. microRNAs

Body fluids contain several types of molecules including circulating tumor cells (CTCs), circulating nucleic acids (both DNA and RNA), and extracellular vesicles (EVs) [23,24,25]. Among those, in particular, circulating microRNAs (miRNAs) have attracted research interest due to their extraordinary stability in body fluids [26]. MiRNAs are small non-coding RNAs (snRNA) that play an important role in gene regulation [27]. miRNAs modulate gene expression by binding a complementary sequence of a target mRNA [28,29]. Of note, a single miRNA can regulate the expression of hundreds of mRNAs and, conversely, an mRNA may present numerous sequences that can guarantee interaction with multiple miRNAs [30]. When taking into consideration the involvement of miRNAs in several biological processes, it is clear that they may play a role in many diseases, including cancer [31,32]. An aberrant miRNA profile is indeed associated with tumor development, progression, metastasis process, and chemotherapy response, suggesting their possible use not only as diagnostic biomarkers but also as predictive biomarkers of therapeutic response [33]. In particular, in recent years, growing interest has been paid to circulating miRNAs, which are detected in body fluid as complexed with other RNA binding proteins or enclosed in EVs. In both cases, the miRNA is protected from enzymatic degradation, ensuring that it can carry out its function [34,35]. Over the years, many studies have identified different miRNAs as potential diagnostic and prognostic biomarkers in EOC. However, in most of these, deregulation was observed when comparing the tumor with a normal counterpart or healthy tissue. On the contrary, the studies that analyzed miRNA expression in relation to pharmacological response are limited and with a small consensus. Given these premises, the aim of this review is to provide a picture of the current knowledge on circulating miRNAs identified to be significantly associated with EOC clinical response. 

## 2. Methods

### Systematic Review of Studies Investigating Circulating miRNAs in Therapeutic Response in EOC Patients

To this purpose, we systematically searched for papers analyzing expression of circulating miRNA in EOC in relation to prognostic and molecular classifications. 

**Figure 1:**
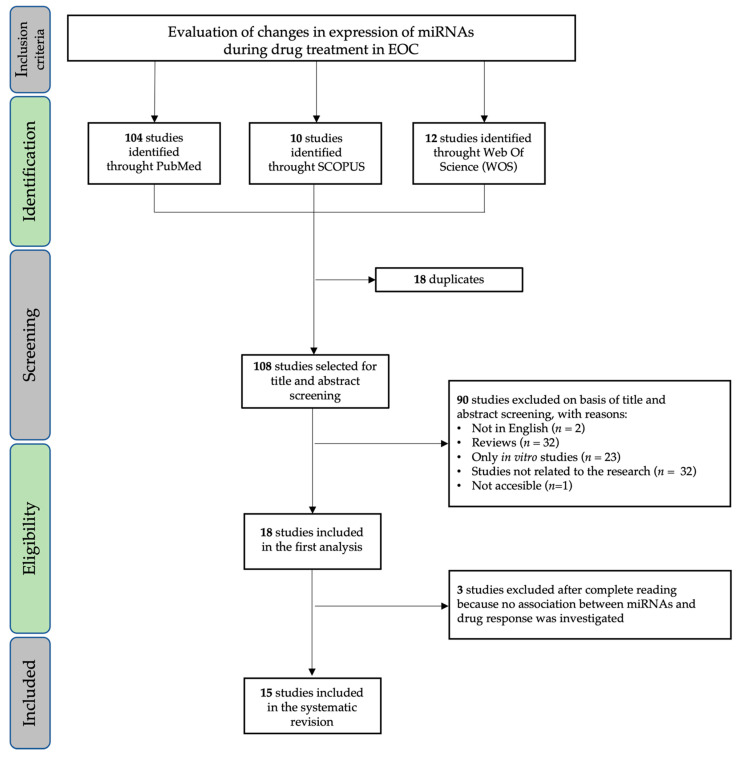
Workflow of the systematic review.

The systematic review was conducted in accordance with the PRISMA Statement principles [36]. The research question was “can miRNAs be used as biomarkers to monitor clinical response in EOC?”, and it was determined using the PICOS process (population, intervention, comparison, outcomes, study design) [37]. PubMed, Web of Knowledge, and Scopus databases were systematically searched for original articles analyzing the circulating miRNAs associated with drug response in EOC (last updated search 1 August 2021). The papers included in this revision are summarized in Table 1. Relevant studies were selected using the Boolean combination of the following key terms: “miR OR miRNA or miRNAs or microRNA” AND “circulating OR plasma OR whole blood OR serum OR ascites OR effusions OR exosome OR exosomes OR exosomal” AND “ovarian cancer OR tumor OR tumour OR neoplasia OR carcinoma OR tumors OR tumours OR cancers OR carcinomas” AND “adjuvant OR neoadjuvant OR clinical response OR chemotherapy OR treatment response”. Additionally, the reference lists of reviews, meta-analyses, and all original studies were hand-searched to acquire further relevant studies missed from the initial electronic search (Figure 1).

Eligible studies were required to meet the following inclusion criteria: studies evaluating circulating miRNAs in relation to therapy in EOC. Exclusion criteria were: (i) meta-analyses, reviews, and editorials; (ii) non-human studies; (iii) in vitro studies; (iv) non-English articles.

After removing duplicate studies, two investigators (GR and FG) independently checked titles and abstracts of the retrieved articles and judged their eligibility. One study was not accessible [38]. Then, the entire text of potentially eligible studies was evaluated to assess appropriateness of inclusion in this systematic review. The same two authors independently extracted the following data from the selected papers: (1) first author, publication year, and aim; (2) sample size; (3) type of drug; (4) type of body fluid (plasma/serum/exosomes), techniques used, and validations; (5) type of association between circulating miRNA and clinical outcome. The results are reported in Table 1. 

**Table 1:** Studies included in the systematic review.

Author, Year, [ref.]	Aim of the Study	Number of Patients	Additional Details and Histology (If Reported)	Therapy	Biological Matrix	Technique/s Used	Validation of the Results	Most Important Findings
**miRNAs expression in chemotherapy resistant and sensitive OC patients**								
Li et al., 2021 [39]	To characterize the expression of hsa-miR-105 in PTX-resistant EOC	105 EOC pts: *n* = 59 resistant, *n* = 56 sensitive to chemo	Primary diagnosis EOC	TX-based chemo	Plasma	qRT-PCR	Cell lines and xenograft models	↓ miR-105 in PTX-resistant EOC, to PTX-responsive EOC (*p* < 0.0001).
Chen et al., 2020 [40]	To investigate serum miR-125b as a biomarker for diagnosis and prediction of treatment response in EOC	83 EOC pts: *n* = 35 resistant, *n* = 48 sensitive to chemo	Primary diagnosis EOC	PT and TX-based chemo	Serum	qRT-PCR	\	↓ miR-125b in PT-resistant EOC pts
Biamonte et al., 2019 [41]	To explore the functional roles of let-7g in EOC	17 EOC pts: *n* = 9 resistant, *n* = 8 sensitive to chemo	Primary diagnosis HGSOC (Stage IIIc–IV)	PT + TX + BVZ	Serum	qRT-PCR	Cell lines	↓ let-7g in resistant EOC pts
Kuhlmann et al., 2019 [42]	To explore the signature of EV-associated miRNAs in PT-resistant EOCs	30 EOC pts: *n* = 15 resistant, *n* = 15 sensitive to chemo	SOC	PT and TX-based chemo	Exosomes from plasma	Illumina NGS	\	12 miRNAs (miR-181a-2-3p, miR-1908-5p, miR-1304-3p, miR-486-3p, miR-21-3p, miR-548o-3p, miR-1185-1-3p, miR-223-5p, miR-664-5p, miR-345-5p, miR-625-3p, miR-443b-3p); however, after adjustment, no significance maintained
Fukagawa et al., 2017 [43]	To identify candidate circulating miRNAs as biomarkers, and potential therapeutic targets	Profiling in 12 EOC pts: *n* = 6 resistant, *n* = 6 sensitive to chemo. Validation in 98 sera	Primary diagnosis EOC (Profiling: *n* = 7 SOC, *n* = 7 EMOC, *n* = 1 CCOC; Validation: *n* = 34 SOC, *n* = 16 EMOC, *n* = 6 MCOC, *n* = 28 CCOC, *n* = 14 other)	PT and TX-based chemo	Serum	Agilent Microarray; qRT-PCR	Independent cohort of pts; cell lines and xenograft models	↑ miR-135a-3p associated with ↑ OS
**Longitudinal analysis of miRNA levels to monitor chemotherapy response**								
Robelin et al., 2020 [44]	To identify specific circulating miRNAs to monitor disease burden and guide clinicians in decision making for EOC pts	Profiling in 8 EOC pts; validation in 111 OC pts	Primary diagnosis EOC (Profiling: *n* = 8 SOC; Validation: *n* = 98 SOC, *n* = 1 EMOC, *n* = 1 CCOC, *n* = 1 MCOC, *n* = 3 Undifferentiated, *n* = 7 NA)	PT and TX-based chemotherapy +/− nintedanib and debulking surgery	Plasma	miScript miRNA PCR Array (Qiagen); qRT-PCR	Independent cohort of pts	The longitudinal kinetics of miRNA expressions were highly inconsistent and there was no relation with the CA-125 dynamics
Zhu et al., 2019 [45]	To analyze the correlation between exosomal miR-223 and recurrence	12 relapsed EOC pts. 2 time points: at the time of surgery and after recurrence	SOC (stage IIIC–V)	PT and TX-based chemo	Exosomes from serum	qRT-PCR	Cell lines and xenograft models	↑ miR-223 at recurrence vs. time of surgery
Kobayashi et al., 2018 [46]	To identify circulating miRNAs as potential diagnostic and prognostic biomarkers in HGSOCs	16 EOC pts. 2 time points: before surgery and after the first post-surgical chemo cycle (about 28 days after surgery)	Primary diagnosis HGSOC	PT and TX-based chemo	Serum	qRT-PCR	Cell lines	↓ miR-1290 after debulking surgery and chemo
Grabosch et al., 2017 [47]	To confirm the feasibility of collecting serial peritoneal samples from implanted catheters in EOC pts receiving IP chemo	13 EOC pts. 3 time points: after surgery, before chemo (T0) and after the first (T1) and second (T2) cycles of chemo	Primary diagnosis EOC (*n* = 9 SOC, *n* = 3 EMOC, *n* = 1 CCOC)	PT + TX + BVZ-based IP chemo	Plasma (*n* = 9) and PW/PF (*n* = 4)	NanoString nCounter miRNA Expression Assay	\	In plasma, T0 vs. T1: 55 miRNAs deregulated; T1 vs. T2: 33 miRNAs deregulated In PW/PF, T0 vs. T1: 12 miRNAs deregulated; T1 vs. T2: 33 miRNAs deregulated
Benson et al., 2015 [48]	To identify alterations in circulating miRNAs associated with decitabine followed by carboPT chemo treatment	14 EOC pts. 2 time points: at baseline and on day 29 after first cycle of chemo	EOC, progressed to previous PT-based chemo	Decitabine followed by carboplatin chemo	Plasma	qRT-PCR miRNA OpenArrays (Thermo)	\	In the overall cohort, T0 vs. T1: ↓ miR-193a-5p and miR-375 after chemotherapy; In the non-responder pts, T0 vs. T1: ↑ miR-339-3p, miR-340-5p, miR-133a, and miR-10a, ↓ miR-375, miR-25-3p, and miR-148b-5p. In decitabine, sensitive vs. resistant pts: ↑ miR-616, miR-532-3p, and miR-148b-5p after first cycle of chemo
Kapetanakis et al., 2015 [49]	To assess the plasma levels of miR-200b in EOCs in a longitudinal study	33 EOC pts: *n* = 9 unresectable tumors treated with chemo, *n* = 14 debulking after chemo, *n* = 10 direct debulking. 2 time points: pre- and post-chemo	HGSOC	PT and TX-based chemo	Plasma	qRT-PCR	\	Pre vs. post-chemotherapy: ↓ miR-200b in 33% of unresectable tumors versus in 54% for tumors resectable immediately or after neoadjuvant chemo
Kuhlmann et al., 2014 [50]	To identify deregulated miRNAs/snRNAs in sera of EOC pts and investigate their potential in therapy monitoring	69 EOC pts. 2 time points: before surgery (*n* = 63) and after post-surgical chemo (*n* = 56)	Primary diagnosis EOC (*n* = 45 SOC, *n* = 5 MCOC, *n* = 5 EOC, *n* = 3 CCOC, *n* = 4 mixed, *n* = 7 other)	PT-based chemotherapy	Serum	Agilent Microarray; qRT-PCR	\	↑ RNU2-1f in pts with residual abdominal tumor mass after chemotherapy and PT resistance. In 50 pts with available paired serum samples before surgery and after adjuvant chemotherapy: pts with persistently RNU2-1f-positive levels had ↓ PFS and OS
Shapira et al., 2014 [51]	To analyze circulating miRNAs as potential biomarkers for EOC detection and outcome	5 EOC pts. 2 time points: before surgery and after post-surgical chemotherapy	Primary diagnosis EOC	PT-based chemotherapy (not clearly indicated)	Plasma	qRT-PCR miRNA OpenArrays (Thermo)	\	↓ miR-1274a, miR-1274b, and miR-1290 after treatment; ↑ miR-19b, miR-25, miR-195, and miR-16 in post-chemotherapy samples
**Association between miRNAs and clinical response**								
Vigneron et al., 2020 [52]	To assess the predictive value of circulating miR-622 prior to first-line chemotherapy and at relapse	130 EOC pts (*n* = 65: prospective cohort, *n* = 65 retrospective cohort; additional *n* = 35 at relapse, from the retrospective cohort)	Newly diagnosed HGSOC (stages III–IV)	PT and TX-based chemotherapy	Serum	qRT-PCR	Independent cohort of pts (prospective and retrospective)	↑ miR-622 in pts with ↓ PFS
Halvorsen et al., 2017 [53]	To identify circulating miRNAs able to identify EOC pts at high risk for relapse	207 EOC pts: Profiling in 91 EOC pts; validation in 116 EOC pts	Primary diagnosis EOC (Profiling: *n* = 58 SOC, *n* = 6 EMOC, *n* = 2 MCOC, *n* = 13 CCOC, *n* = 8 mixed, *n* = 4 other; validation: *n* = 79 SOC, *n* = 6 EMOC, *n* = 0 MCOC, *n* = 14 CCOC, *n* = 13 mixed, *n* = 4 other)	PT and TX or PT and TX-based chemotherapy + BVZ	Plasma	Taqman miRNA low density array (Thermo); qRT-PCR	Independent cohort of pts	↓ miR-200c in pts with ↑ OS treated with BVZ

BVZ: bevacizumab; Chemo: chemotherapy; CCOC: clear cell ovarian carcinoma; EOC: epithelial ovarian cancer; HGSOC: high-grade serous ovarian cancinoma; EMOC: endometrioid ovarian carcinoma; IP: intraperitoneal; MCOC: mucinous ovarian carcinoma; PF: peritoneal fluid; PW: peritoneal washing; pts: patients; PTX: paclitaxel; PT: platinum; SOC: serous ovarian carcinoma; TX: taxane; ↑: higher; ↓: lower; \: information not available.

## 3. Results

**Figure 2:**
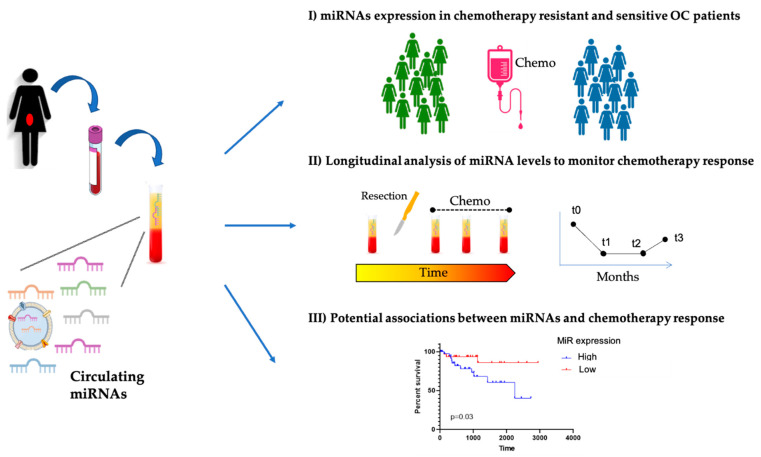
Main types of studies investigating circulating miRNAs and therapeutic response in EOC.

We included in the final review a total of 15 works. The majority of the studies analyzed miRNAs in plasma or serum, a small portion (*n* = 3) investigated exosomal miRNAs, and one analyzed peritoneal washing (PW) and fluid (PF). Overall, the studies retrieved can be divided in three different groups based on the main goal (Figure 2): (i) comparing miRNA expression in chemotherapy-resistant and -sensitive OC patients; (ii) longitudinal analysis of miRNA levels to monitor chemotherapy response; (iii) identifying potential associations between miRNAs and chemotherapy response (i.e., in terms of progression-free survival (PFS) or overall survival (OS)).

### 3.1. miRNA Expression in Chemotherapy-Resistant and -Sensitive EOC Patients

The first study analyzing the expression of circulating miRNAs in chemotherapy-sensitive and -resistant EOC patients was published in 2017 [43]. Resistance was defined as relapse occurring ≤6 months following the completion of chemotherapy. The authors first analyzed the miRNAs’ global expression profile in 12 EOC patients, of which six were platinum-resistant (had recurrence within 6 months after completion of platinum and taxane-based treatment) and six showed platinum sensitivity. Based on this comparison, the authors identified three deregulated miRNAs (miR-135a-3p, miR-630, and miR-1207), which were further validated in 98 EOC sera. In particular, after having stratified the patients based on the median value for each miRNA, they showed that EOCs with higher miR-135a-3p had significantly improved OS compared to the patients with lower miRNA levels. To provide clinical insights in EOC, miR-135a-3p expression in sera was compared with the one in peritoneal fluid and tissue samples of patients with EOC, ovarian cysts, normal ovaries, or endometrial cancer. In all these comparisons, the biological matrix related to EOC patients showed lower miR-135a expression. Finally, functional studies demonstrated that in OC cell lines (SKOV-3 and ES-2), enhanced miR-135a-3p expression was able to promote cisplatin and paclitaxel sensitivity and suppress cell proliferation and xenograft tumor growth. Subsequently, Kuhlmann et al. evaluated the exosomal miRNAs in 30 EOC patients by Illumina NGS [42]; among those, 15 patients recurred within 6 months after the adjuvant platinum-based chemotherapy, whereas 15 remained platinum-sensitive. In addition, the authors compared different EV-enrichment strategies for optimizing the miRNA isolation and library preparation. The results showed the deregulation of 12 miRNAs (hsa-miR-181a-2-3p, hsa-miR-1908-5p, hsa-miR-1304-3p, hsa-miR-486-3p, hsa-miR-21-3p, hsa-miR-548o-3p, hsa-miR-1185-1-3p, has-miR-223-5p, hsa-miR-664-5p, hsa-miR-345-5p, hsa-miR-625-3p, and hsa-miR-443b-3p); however, after adjustment, the findings did not maintain statistical significance. However, the results are of potential interest considering that among these miRNAs, a few (miR-181a, miR-1908, miR-21, miR-486, and miR-223) were previously reported in EOC [54,55,56,57]. Besides these two papers investigating large profiles of miRNAs, the other works published in the literature explored single miRNAs from previous evidence on different cancer types. Biamonte and colleagues explored the role of let-7g in EOC and chemoresistance. The analysis started from an in vitro evaluation in two OC cell lines showing that let-7g acts as a tumor suppressor in EOC and that its enhanced expression promotes higher sensitivity to cisplatin treatment. To further corroborate the results, let-7g levels were evaluated in the tissue and serum of 17 EOC patients, highlighting that in both cases let-7g was expressed at a significantly lower level in chemotherapy-resistant cases (*n* = 9) compared to chemo-sensitive cases (*n* = 8). Another example of a single miRNA investigated in EOC and chemoresistance is miR-125b. This miRNA was previously characterized in EOC specimens as markedly poorly expressed [58,59], but its correlation with therapeutic response had not been investigated. In a recent work, Chen and colleagues [40] first compared circulating miR-125b in sera from EOC (*n* = 152), healthy controls (*n* = 42), and benign and borderline tumors (*n* = 30 and *n* = 35, respectively) and confirmed that lower levels were detected in EOC patients. In this cohort of EOC cases, miR-125b was also correlated with FIGO stage and lymph node metastasis. With regard to chemotherapy resistance, the authors showed that sensitive patients had miR-125b upregulation compared to the non-sensitive patients. More recently, Li et al. deepened the understanding of the role of miR-105 in EOC starting by data mining publicly available datasets comprising the miRNA profiling of EOC cells and their PTX-resistant sublines [39]. Based on that, miR-105 was significantly downregulated in PTX-resistant cell lines compared to parental ones, and this deregulation was further confirmed by the same authors by generating a set of two additional PTX-resistant models (exposing PTX-sensitive cells to increasing doses of PTX) and their matched xenograft models. In both cells and xenografts, lower miR-105 expression was significantly associated with PTX resistance. To further test these findings, tissue and sera clinical specimens from 105 EOC patients were analyzed. The results revealed that miR-105 was significantly decreased in both tissue and sera derived from PTX-resistant patients compared with the PTX-responsive cases. With regard to the circulating miR-105 in particular, high plasmatic levels were associated with improved responsiveness to PTX. All the studies applied the same 6-month cut off to define resistance.

### 3.2. Longitudinal Analysis of miRNA Levels to Monitor Chemotherapy Response

Longitudinal analysis of miRNA levels, through the collection of multiple blood samples over time, is particularly interesting because it may display the peculiar deregulation of certain miRNAs potentially correlated with poor or good response to specific drugs, including chemotherapy.

The first studies in EOC with this purpose analyzed a small sample size of patients. 

Shapira et al. investigated plasma samples of 42 EOC patients; however, the association between miRNAs and therapeutic response was evaluated in only five cases, with OS > 4 years, for whom blood samples were collected both before surgical resection and after chemotherapy [51]. The comparison showed seven differentially expressed miRNAs between presurgical and post-chemotherapy time points; in particular, miR-1274a, miR-1274b, and miR-1290 were decreased after treatment, whereas miR-19b, miR-25, miR-195, and miR-16 displayed over-expression in post-chemotherapy samples. Comparison between plasma collected before and within 2 weeks from the surgical resection did not show any difference. Similarly, Kuhlmann and colleagues started their analysis by profiling miRNA expression in five EOC patients and five healthy controls [50]; based on the results, one snRNA, *RNU2-1f*, was selected for further validation in 69 sera, of which *n* = 63 were collected before surgery and *n* = 56 after adjuvant platinum-based chemotherapy. The detection of *RNU2-1f* within the profiling was made by two probes (miR-1246 and miR-1290) that were previously shown to be specific for *RNU2-1* since they detect fragmented forms of *RNU2-1* [60,61]. While the expression of *RNU2-1* was confirmed to be higher in EOC patients versus the healthy controls (independently by the specific time points), no differences were observed between preoperative circulating *RNU2-1f* and after adjuvant regimen. Interestingly, for a subset of 15 patients with suboptimal primary debulking, radiographic reports on restaging after chemotherapy were available; of these, 10 were defined platinum-sensitive and five resistant. The levels of *RNU2-1f* were significantly higher in patients with residual abdominal tumor mass after chemotherapy and platinum resistance. Finally, for 50 patients, for whom paired serum samples before surgery and after adjuvant chemotherapy were available, *RNU2-1f* abundance dynamics were evaluated. Kaplan–Meier analysis highlighted that the patients who had persistently *RNU2-1f*-positive levels at primary diagnosis and after chemotherapy showed significantly shorter PFS and OS than the other patients. 

Kapetanakis et al. evaluated the expression of miR-200b in 33 patients, with blood samples collected before a diagnostic laparoscopy and at the end of the primary treatment (treatment including chemotherapy and debulking surgery when feasible), 4-8 months after the initial laparoscopy [49]. The authors also evaluated the association between miR-200b and the serum marker CA-125. CA-125 levels returned to normal plasma concentrations within the first months of the treatment, even among patients with unresectable tumors. On the contrary, expression levels of miR-200b were quite heterogeneous among the different types of EOCs. In general, the proportion of patients with decreasing concentrations of miR-200b was 33% for unresectable tumors versus 54% for patients with resectable tumors treated with adjuvant or neoadjuvant chemotherapy. For 24 out of 33 EOCs, follow-up longer than 10 months was available and the miR-200b level was analyzed in association with PFS. Patients with a miR-200b-negative variation pre- and post-chemotherapy showed significantly longer PFS, compared with the remaining patients, even after adjustment for multiple variables. All these data, taken together, suggest that specific miRNAs could be more sensitive liquid biomarkers than CA-125, which is currently widely used in clinical management. Similarly, Kobayashi et al. evaluated miR-1290 with a longitudinal approach [46]. The work originated from an miRNA profiling from in vitro models of OC and normal ovary cells, which was then validated in clinical specimens, confirming a higher expression of miR-1290 in EOC patients compared with healthy control sera. The same miRNA was also evaluated before and after the first cycle of adjuvant chemotherapy in 16 patients. In line with the other results, miR-1290 expression was significantly decreased after debulking surgery and chemotherapy, suggesting that circulating miR-1290 may be directly related to tumor burden. Similarly, Zhu and collaborators characterized the role of exosomal miR-223 in chemoresistance, starting with a careful in vitro study in cell lines and xenograft models [45]. The authors demonstrated that exosomal miR-223 derived from macrophages was able to foster drug resistance in EOC cells and that its upregulation is directly associated with a chemoresistant phenotype. To further test this hypothesis, the authors compared sera of 12 patients collected before and after resistance occurrence, confirming an increased miR-223 expression at the time of recurrence.

More recently, Robelin et al. published a longitudinal report including a large number of patients (*n* = 119), which is, so far, the widest series investigated [44]. The enrolled patients received standard neoadjuvant and adjuvant chemotherapy (three to four cycles) before and after cytoreductive surgery, followed by a maintenance treatment with nintedanib/placebo for up to 2 years. In total, the authors were able to assess 756 serial blood samples. From a profiling of 84 miRNAs in eight patients, and from literature data, 11 miRNAs (iR-15b-5p, miR-16-5p, miR-20a-5p, miR-21-5p, miR-93-5p, miR-122-5p, miR-150-5p, miR-195-5p, miR-200b-3p, miR-148b-5p, and miR-34a-5p) were selected to be further tested in serial blood samples derived from 111 EOC cases. However, even with the good clinical design of the study, the results were mainly negative; indeed, as clearly highlighted by the same authors, the longitudinal kinetics of the 11 miRNA expressions were highly inconsistent, and no relation with CA-125 dynamics was identified. The miRNA changes during neoadjuvant treatment were not found to be associated with RECIST tumor response or outcomes. The conclusion of this study indicates, therefore, a lack of assessable longitudinal prognostic or predictive kinetic profiles for the selected miRNAs, which cannot be automatically applied to other miRNAs.

Finally, among the papers investigating circulating miRNAs and therapeutic response, two are different but deserve to be included in this list. In particular, the first, by Benson et al., evaluated plasmatic miRNA levels in EOC patients treated with a regimen of low dose decitabine—a DNA methyltransferase inhibitor—and carboplatin [48]. The second, published by Grabosch and colleagues, investigated circulating miRNAs in serial peritoneal samples in women receiving intraperitoneal (IP) chemotherapy [47]. The analysis by Benson and collaborators included 14 EOC patients enrolled in the previously described open label phase II clinical trial [62]. This study is particularly relevant because, among the works herein described, it is the only one focused on platinum-resistant, recurrent patients treated with an alternative drug. The aim of this report was to characterize the alterations in circulating miRNAs associated with decitabine followed by a carboplatin chemotherapy regimen and clinical response. To this purpose, plasma samples were collected before treatment and after the completion of the first cycle of treatment (day 29). Among the 14 patients, *n* = 8 showed tumor progression prior to six cycles of chemotherapy and were considered non-responders, whereas the remaining six were considered responders. By simultaneously analyzing 93 miRNAs, the authors identified 10 miRNAs related to response to decitabine followed by carboplatin chemotherapy. In detail, miR-193a-5p and miR-375 decreased after chemotherapy; moreover, in the non-responder patients, four miRNAs (miR-339-3p, miR-340-5p, miR-133a, and miR-10a) displayed increased levels, while three miRNAs (miR-375, miR-25-3p, and miR-148b-5p) showed a significant decrease. MiRNA expression was compared also in resistant and sensitive patients at the post-treatment timepoint; in this regard, the authors observed three miRNAs (miR-616, miR-532-3p, and miR-148b-5p) that were significantly increased in responders. Finally, Kaplan–Meier analysis was applied to evaluate if any of the miRNA alterations were able to predict treatment response. In this case, four patients were excluded due to their progression within the first cycle of chemotherapy. The remaining 10 were divided into two groups (high and low expression) based on the median value of each miRNA, showing that a lower concentration of miR-148b-5p on day 29 was associated with disease progression.

The second previously mentioned study aimed to assess miRNA expression in serial peritoneal samples from implanted catheters in women receiving IP chemotherapy. The analysis involved 13 women, and, besides miRNAs, other potential biomarkers were evaluated, including, but not limited to, immune genes and cytokines. miRNAs were profiled in plasma (*n* = 9), peritoneal fluid (PF, *n* = 1), and peritoneal wash (PW, *n* = 3) at three time points (T0: after surgery, before chemo; T1: after the first cycle of chemo; T2: after the second cycle of chemo) using the NanoString nCounter miRNA Expression Assay. In plasma, after the first round of chemo (T0 vs. T1) and after the second cycle of chemotherapy (T1 vs. T2), 51 and 33 miRNAs were deregulated, respectively, eight of which were in common. When altered, the miRNA tended to remain expressed in the same direction (up or downregulated from baseline). On the contrary, in PW, a larger number of miRNAs were deregulated after the second cycle of chemo (T0 vs. T1: 12 miRNAs; T1 vs. T2: 33 miRNAs). As suggested by the authors, plasma miRNAs may be modulated by early changes due to systemic effects of chemotherapy. In contrast, PW miRNAs can be related to later local tumor changes. Interestingly, observing the deregulated miRNAs in plasma and PW, no overlap was detected, implying that the alterations of miRNAs happening in PF/PW (at local level) could be not detected by analyzing plasma miRNAs. In this context, PF/PW evaluation could be particularly appealing to accurately monitor molecular changes, assess response to therapy, or to develop more personalized therapeutic approaches.

### 3.3. Association between miRNAs and Clinical Response 

The first work to assess the association between miRNAs and clinical response—in terms of PFS or OS—in EOC dates to 2017 [53]. Specifically, Halvorsen et al. enrolled 207 EOC patients, under standard chemotherapy or in association with bevacizumab, aiming at identifying circulating miRNAs able to discriminate patients at high risk for relapse. The discovery step assessed the levels of 754 miRNAs in 91 sera. The remaining 116 patients were included in the validation cohort; patients were stratified based on treatment type and survival length (in long or short PFS). Four miRNAs (miR-1274a, miR-141, miR-200b, and miR-200c) were shown to be significantly associated with survival. In the validation set, miR-141 and miR-200b confirmed the prognostic association. Considering the treatment, no difference in PFS related to miRNAs was observed in the discovery set; however, in the validation set, low levels of miR-200c were associated with significantly better survival in patients treated with bevacizumab (with 5-month prolongation of PFS) compared to standard chemotherapy. No additional associations were reported. Vigneron et al. analyzed the ability of miR-622 to predict platinum response [52].

This miRNA has been reported to be involved in the homologous recombination repair system, which plays a role in the platinum mechanism of action [63,64]. The authors analyzed miR-622 in two distinct cohorts of 65 HGSOC patients (one prospective and one retrospective) treated with adjuvant platinum and taxane-based chemotherapy. The sera were collected before the first cycle of chemotherapy; moreover, for 35 patients included in the retrospective cohort, an additional serum sample was available at the time of relapse. Each cohort was sorted into miR-622 low and high expression based on a cut-off value. In the prospective group, the high expression of miR-622 group was associated with significantly lower PFS compared with the patients showing lower miR-622 levels; similarly, high miR-622 expression was correlated with lower OS. In the validation, in the retrospective cohort, applying the same cut-off value, high miR-622 expression was correlated with lower OS; however, in the multivariate analysis, this did not maintain statistical significance. With regard to the predictive value at relapse, the 35 patients were divided into short-term (<12 months) and long-term (>12 months) survivors according to the OS and a new cut-off value was calculated by an ROC curve. Once again, high miR-622 levels were correlated with lower OS compared with patients with lower miR-622. The new cut-off value was re-applied to the retrospective cohort, and this time the correlation of high miR-622 expression/lower OS maintained statistical significance even in the multivariate analysis. All these results together showed that miR-622 was an independent predictive factor of PFS and OS in the prospective cohort, prior to first-line chemotherapy; in the retrospective cohort, miR-622 was a predictive factor of OS before first-line chemotherapy and at the time of relapse.

## 4. Discussion

Ovarian carcinoma is one of the most lethal cancers worldwide; this is mostly due to its unspecific symptoms and the lack of screening tests, which, taken together, contribute to delaying diagnosis and treatment. The current serum biomarker, CA-125, lacks sensitivity and specificity. It is useful in identifying primary and relapsed disease and correlates with disease burden, but is inadequate in the response to chemotherapy and risk of relapse [10,11]. 

Given that, the identification of novel biomarkers able to foster more precise medical approaches and the personalization of patients’ management represents an unmet clinical requirement. In this context, circulating miRNAs may represent an interesting opportunity as they are highly stable and can be easily detected in all biological fluids, including blood samples. This is particularly relevant when looking for non-invasive approaches that can be repeated over time, with no pain and stress for the oncological patient. Based on this, it is reasonable to think that miRNAs could potentially be integrated into the existing prognostic outline and promote a better patient management. In this regard, the present review aimed to describe the circulating miRNAs currently reported as associated with therapeutic treatments in EOC. Considering that most of reports have investigated tissue miRNAs, the available literature results are limited and we were able to identify only 15 studies focused on our topic. Among those, the majority analyzed serum/plasma miRNAs, three exosomal miRNAs, and one evaluated PF/PB. Eight of 15 used large profiling to simultaneously screen multiple miRNAs, whereas the remaining adopted RT-PCR as the main technique to evaluate a limited number of miRNAs; four studies had an independent cohort of patients to validate their preliminary findings and five described functional validations in cell lines and/or animal models. 

Overall, as previously mentioned, the available reports can be divided according to their main goals, thus identifying three main groups (Figure 2); however, even considering the specific aims, the consensus among the studies remains very limited. With all aspects taken together, it is understandable that no clinical translation has happened, and it seems that further extensive research will be needed to define reliable miRNAs as candidate biomarkers. In addition, the lack of standardized protocols, including sample collection, the type of biological fluid, RNA extraction, and techniques, makes it challenging to compare the results between independent studies. We should also be aware that it would be particularly difficult to identify one or a few miRNAs that are able, by themselves, to accurately monitor therapeutic response in EOC patients based on molecular or clinical features. The best approach would be combining multiple variables (including, but not limited to, miRNAs, any DNA mutations, and clinical parameters).

Recently, advances in therapeutic monitoring in EOC have been made with circulating tumor DNA (ctDNA) providing important evidence about its utility in determining outcome and individualizing cancer therapy in patients with EOC [65,66,67]; on the contrary, the role of circulating miRNAs in EOC clinical monitoring needs to be further investigated in order to obtain a larger concordance between the results from independent investigators. Of note, we should bear in mind that ctDNA represents a sort of barcode originating directly from the tumor, but “liquid” miRNAs are not derived uniquely from the cancerous mass. Indeed, miRNAs are also physiologically released by other, normal cells and this makes the general landscape more complex to decipher. As a consequence, the research on cancer liquid biomarkers is still in its embryonal phase and no reliable miRNA candidates to accurately follow the treatment response “in real-time” have been identified yet.

Based on the data reported in our work, the most appealing miRNAs in EOC belong to the miR-200 family. Indeed, two independent works have identified miR-200b and miR-200c as potential biomarkers. Given that, the miR-200 family could have a role as a non-invasive biomarker in EOC. This family has already been reported as of potential interest in gynecological cancers, particularly in endometrial cancer [68]. The above-mentioned correlation could be due to the involvement of miR-200s in the epithelial–mesenchymal transition (EMT) process, which is known to play a key role in EOC progression, metastases, and recurrence and to be one of the cancer escape routes to medical treatments [69].

## 5. Conclusions

So far, the role of circulating miRNAs in therapeutic monitoring in EOC remains to be clarified given the inconsistent findings reported by different studies. This could be in part due to the limited number of analyses, the small sample size, and the lack of a standardized procedure to properly assess the miRNAs’ contribution. Nevertheless, circulating miRNAs have potential as novel non-invasive and highly useful biomarkers in EOC.

Further studies with standardized protocols and larger cohorts of patients are warranted to foster the identification of circulating miRNAs of potential clinical significance in EOC.
